# Keeping Proteins on Target

**DOI:** 10.1371/journal.pbio.0020361

**Published:** 2004-09-21

To keep a cell healthy, proteins must go to the right places within a cell. To direct proteins to specific areas of the cell, they're marked with tags akin to zip codes on mail. These tags can direct one protein to the cell's nucleus, where it regulates gene expression, say, while another is sent to the hinterlands of the cell membrane, where it receives environmental signals. Protein targeting is crucial even as cells are building proteins—otherwise, for example, proteins won't fold into the proper shape.

There's a pair of proteins that, across all organisms, plays a key role in protein targeting. Called Ffh and FtsY in bacteria, these proteins each have an active and inactive state; only when they're in their active state can they bind each other and deliver other proteins to their proper location. Both these proteins are GTPases, a class of proteins that work as molecular switches. However, among GTPases, Ffh and FtsY are unique. Other GTPases switch between active and inactive states by binding different forms of a small, energy-carrying molecule—either GTP or GDP. Such GTPases often require the help of other proteins to switch states. Ffh and FtsY, on the other hand, almost always have GTP bound. And when they interact, they change each other's state—allowing each other to convert GTP to GDP—without the help of other proteins. But researchers didn't know exactly how these proteins interacted or how they switched between their active and inactive forms.

Now, as reported in this issue of *PLoS Biology,* Shu-ou Shan and colleagues have found that when Ffh and FtsY bind and then activate each other, they likely go through an unusual, multi-step process in which the proteins change shapes, flexing so that different parts of the proteins become active at each step.

The researchers mutated 45 different sites in the gene that encodes the FtsY protein to produce a bevy of mutant proteins with different properties. The researchers chose mutations that produced amino acid substitutions at sites in the FtsY protein that have been preserved through evolution, and so are presumably crucial for the protein's function. These sites, it turns out, are all on the protein's surface where it interacts with Ffh.

The mutant versions of FtsY varied in how well they bound to the normal version of Ffh and how quickly the two proteins activated each other. The researchers were able to sort the different mutations into four different classes based on the type of problem the proteins had: some bound Ffh very loosely, some bound Ffh well but only weakly turned on its GTPase activity, and so on. All of these mutations would presumably foul up the protein targeting system, so this explains why certain amino acids have been preserved through evolution.

Both Ffh and FtsY change shapes as they bind, activate each other's GTPase activity, then cleave GTP and release each other, the researchers infer. They don't have direct evidence for these shape changes, but the postulated bends and twists during interaction are consistent with the build of the proteins. These shape changes, they speculate, could switch different parts of each protein between active and inactive states. By showing how this unique type of GTPase switch likely works, Shan and colleagues have helped explain how cells target proteins to specific areas—and perhaps have paved the way for others to find similar switches elsewhere within cells.

